# Optimizing Sowing Date and Nitrogen Management to Trade Off Yield and Nitrate Leaching in Maize-Soybean Intercropping Under CMIP6 Climate Scenarios in the North China Plain

**DOI:** 10.3390/plants15111753

**Published:** 2026-06-04

**Authors:** Xiaoli Niu, Zhen Yang, Jie Zhang, Xiaoqing Sun, Zhandong Liu, Shihao Jin, Jiaxing Cai, Bingwu Zhang, Yunyan Sun

**Affiliations:** 1 College of Agricultural Equipment Engineering, Henan University of Science and Technology, Luoyang 471000, China; 18336263546@163.com (Z.Y.); 18292735983@163.com (J.Z.); 18832739227@163.com (X.S.); mingy0009@163.com (S.J.); 15903072260@163.com (J.C.); 18237920353@163.com (B.Z.); 17656202677@163.com (Y.S.); 2 Institute of Farmland Irrigation, Chinese Academy of Agricultural Sciences, Xinxiang 453003, China

**Keywords:** climate adaptation, GCM screening, nitrate leaching, biological nitrogen fixation, deep-layer nitrate, sustainable agriculture

## Abstract

Climate change threatens nitrogen cycling in agricultural ecosystems. Optimizing sowing dates and nitrogen management for maize–soybean intercropping is critical for sustainable production in the North China Plain (NCP). Using a calibrated Agricultural Production Systems Simulator (APSIM) model driven by three representative global climate models (GCMs) selected from 20 Coupled Model Intercomparison Project Phase 6 (CMIP6) GCMs, we evaluated management strategies under two Shared Socioeconomic Pathway scenarios (SSP2-4.5 and SSP5-8.5) across three climatic zones for near-term (2030–2059) and long-term (2070–2099) periods. Under SSP5-8.5, warming was 1.8–2.2 times greater than under SSP2-4.5, nitrate nitrogen (NO_3_^−^-N) leaching increased by 12.1%, and nitrate storage in the 100–150 cm soil layer rose by 53.4% in Zone III. Biological nitrogen fixation contributed 20.1–29.1% of soybean nitrogen uptake under low nitrogen and 14.9–23.4% under medium nitrogen. Optimal strategies were identified: sowing on 7 June (S3) with medium nitrogen (220.8 kg N ha^−1^) under SSP2-4.5, and advancing sowing to 28 May (S2) with medium nitrogen under SSP5-8.5 to alleviate heat stress. This study reveals a climate-driven “earlier supply–shortened demand–concentrated leaching” mismatch, providing adaptive management guidance for maize–soybean intercropping systems in the NCP.

## 1. Introduction

The North China Plain is the most important summer maize-soybean production area in China, with 16 million hectares of cultivated land, accounting for about 16% of the national total. For a long time, it has been under the dual pressure of ensuring national food security and controlling nitrate pollution in groundwater [1,2,3]. With the intensification of global climate change, this intensive agricultural system is facing unprecedented challenges. The latest Coupled Model Intercomparison Project Phase 6 (CMIP6) predictions show that in the future, this region will not only experience significant and continuous warming but also witness drastic changes in the temporal and spatial distribution of precipitation, with a significant increase in precipitation variability [4,5]. Climate warming will not only accelerate the mineralization of soil organic nitrogen but also shorten the growth period of crops; the increase in future precipitation variability will greatly increase the risk of deep soil nitrate nitrogen (NO_3_^−^-N) leaching [6]. Therefore, achieving yield–environment trade-offs—maintaining high crop yields while strictly controlling deep soil nitrate leaching under long-term climate change—is an urgent scientific challenge. This is central to current farmland water and nitrogen management.

Intercropping, as a traditional and efficient planting system, enables the intensive utilization of land, labor, soil nutrients, and water-heat resources in both time and space through the combined planting of maize and soybeans [7,8]. Numerous studies have pointed out that the land equivalent ratio of soybean-maize intercropping ranges from 1.26 to 1.81, which can significantly improve resource utilization efficiency and is widely regarded as a sustainable agricultural model for addressing climate change [9]. However, existing studies have deeply analyzed the competition and utilization of aboveground light resources in intercropping systems [10], the advantages of yield formation and their regulatory mechanisms [11], as well as the effects of single temperature or precipitation factors on crop growth, development and yield [12], but few studies have quantitatively analyzed the deep soil nitrogen dynamics and nitrate leaching risks in intercropping systems under future complex climate scenarios. There are complex root interactions in the underground part of intercropping systems, which jointly affect the distribution of soil water and nutrients [13]. Studies have shown that when maize and soybeans are intercropped, maize absorbs a large amount of mineral nitrogen and has a competitive advantage, reducing soil nitrogen content and alleviating the inhibition of soybean nitrogen fixation, which is known as the “mitigation effect” of “nitrogen inhibition” [14]. The main reason is that maize competitively absorbs ammonium and nitrate in the soybean rhizosphere. When soil nitrogen content is low, soybeans meet their nitrogen demand through root nodule nitrogen fixation, thereby increasing nitrogen fixation capacity [15]. Under the background of rising temperatures and changing precipitation patterns in the future, this complex water-nitrogen coupling relationship will shift significantly [16], thereby changing the interspecific “nitrogen transfer” and “nitrogen inhibition” effects and making the risk of deep soil nitrate leaching more severe than that in monoculture systems. Due to these complex interspecific interactions, conclusions from monoculture studies cannot be directly extrapolated to intercropping systems. Therefore, there is an urgent need to clarify the belowground nitrogen environmental risks of intercropping systems under future climate change.

To address the dual risks posed by climate change, adopting proactive adaptive agronomic management is the key to maintaining system resilience. Among them, adjusting sowing dates and optimizing nitrogen application rates are effective management measures for regulating the water-nitrogen coupling process in maize-soybean intercropping systems [17,18]. As an important phenological adaptation strategy, adjusting sowing dates can optimize the crop growth process, offset the shortened growth period caused by climate warming, and match the critical period of crop water and fertilizer demand with future precipitation patterns [19]. Studies have shown that changes in the interspecific symbiotic period caused by differences in sowing time directly alter the competitive and complementary relationship between maize and soybeans for soil water and nitrogen resources, thereby regulating the water-nitrogen coupling process of the system [20]. Nitrogen management is the key to balancing the yield benefits and environmental risks of intercropping systems [21]. Studies have shown that nitrogen supply levels can directly regulate water and nitrogen use efficiency and dry matter accumulation in intercropping systems, thereby affecting system productivity and nitrogen environmental risks [22]. An appropriate nitrogen application rate can not only meet the nitrogen demand for high maize yields but also avoid the inhibitory effect of high nitrogen on soybean nodule nitrogen fixation. A reasonable nitrogen application threshold is also an important prerequisite for controlling deep soil nitrate leaching under variable precipitation. Phenological differences caused by changes in sowing time will reshape the spatiotemporal nutrient demands of crops. Regulating sowing time or nitrogen application rate alone is difficult to achieve the optimal effect, and it is urgent to clarify the coordinated regulatory mechanism of the two. However, the nonlinear interaction process of “gene-environment-management (G × E × M)” driven by long-term climate is complex and difficult to fully quantify through short-term field experiments with limited spatiotemporal scales.

Therefore, advanced agroecosystem modeling has become an indispensable assessment tool for analyzing this complex process. The Agricultural Production Systems Simulator (APSIM) can accurately characterize above- and below-ground resource competition, crop growth mechanisms, and long-term soil water and nitrogen cycling processes in intercropping systems [23,24]. Combined with the high-resolution multi-scenario projection data provided by CMIP6 [25], this study aims to deeply analyze the dynamic evolution of soil nitrogen in maize-soybean intercropping systems in the North China Plain under climate change. The specific research objectives are as follows: (1) Quantify the long-term driving effects of different future climate scenarios (SSP2-4.5 and SSP5-8.5) on the yield stability and deep soil nitrate leaching of intercropping systems; (2) Evaluate the synergistic effects of different sowing times and nitrogen application rates in regulating system nitrogen fixation, nitrogen uptake and environmental emissions; (3) Determine the optimal management strategies that can maximize yield-environment trade-offs under different future climate scenarios. The results of this study will greatly exploit the climate adaptation potential of intercropping systems and provide a quantitative scientific basis and decision support for formulating sustainable agricultural adaptation policies in the North China Plain and similar ecological regions worldwide.

## 2. Results

### 2.1. Future Climate Projections and Driving Factors

#### 2.1.1. Spatiotemporal Trends of Temperature and Precipitation

**Figure 1:**
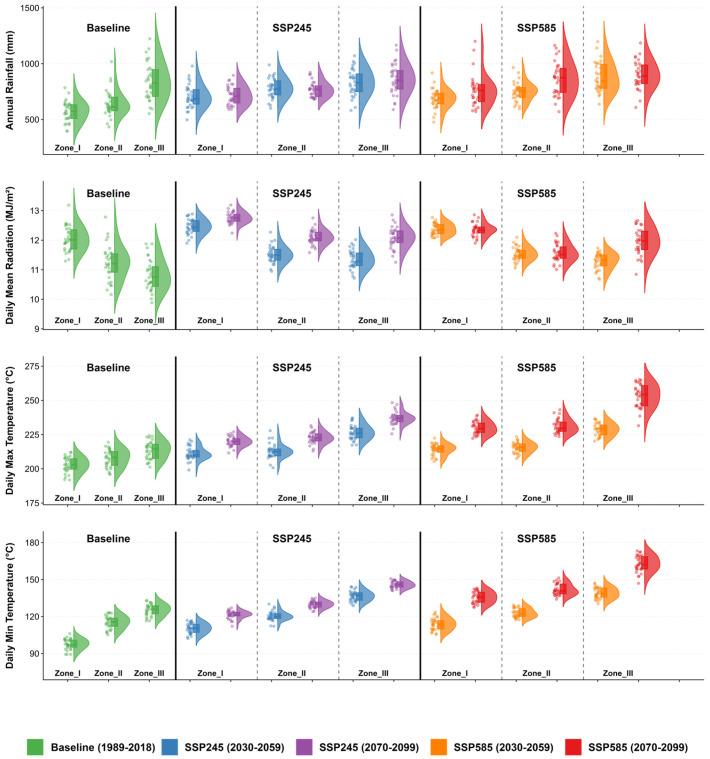
Regional variations in key meteorological factors under different climate scenarios and periods. Annual rainfall, daily mean radiation, daily maximum temperature, and daily minimum temperature across Zone I, Zone II, and Zone III under the Baseline (1989–2018), SSP2-4.5 (2030–2059 and 2070–2099), and SSP5-8.5 (2030–2059 and 2070–2099) scenarios. Violin plots illustrate the probability distribution of each meteorological variable, with overlaid scatter points representing individual data records.

The core characteristics of future climate evolution are shown in Figure 1. Long-term warming is the most prominent common feature of the future climate. Compared with the historical baseline period (1989–2018), the daily maximum temperature in the study area during 2070–2099 under the SSP5-8.5 scenario increased by 2.3–2.5 °C, and the daily minimum temperature increased by 2.1–2.6 °C, with a warming magnitude approximately 1.8–2.2 times that of the SSP2-4.5 scenario. From the perspective of temporal evolution, under the SSP5-8.5 scenario, the daily maximum temperature during 2070–2099 increased by an additional 1.3–1.6 °C compared with the 2030–2059 period, indicating a clear cumulative warming effect. Regionally, the increase in minimum temperature in Zone III (2.6 °C) was higher than that in maximum temperature (2.3 °C), exhibiting asymmetric warming characteristics. In Zone I, the daily mean radiation increased from 12.0 MJ m^−2^ to 12.4 MJ m^−2^ over the same period, an increase of 3.3%.

The core feature of precipitation evolution is a significant increase in interannual fluctuation. Under the SSP5-8.5 scenario, the standard deviation of annual precipitation in each zone increased by 64–94% during 2070–2099 relative to the baseline period. In Zone III, the standard deviation increased from 183.7 mm to 301.5 mm, an increase of 64.1%, making it the zone with the most significant increase in variability. During the same period, the standard deviation in Zone I increased from 179.8 mm to 294.5 mm, an increase of 63.8%, although its mean annual precipitation (785.7 mm) was 14.1% lower than that of Zone III (915.2 mm), exhibiting regional characteristics of “relatively high temperature and low humidity.” From the perspective of temporal evolution, under the SSP5-8.5 scenario, the standard deviation of precipitation in each zone during 2070–2099 increased by 19.3–28.4% compared with the 2030–2059 period, indicating that precipitation instability continues to intensify over time.

#### 2.1.2. Identification of Climate Drivers for Nitrate Leaching

**Figure 2:**
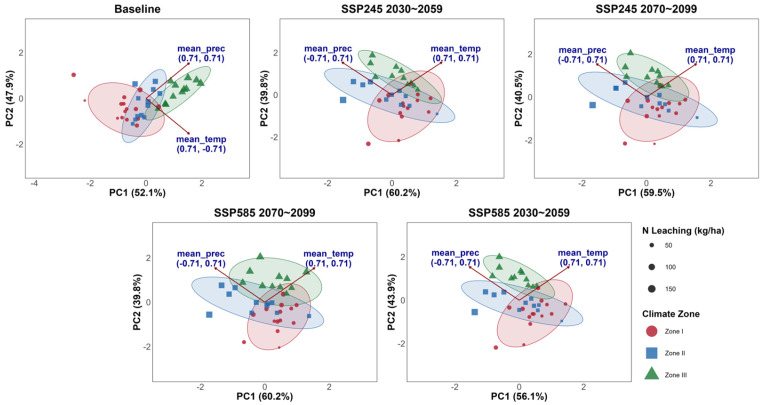
Principal component analysis (PCA) of meteorological factors and nitrate leaching across different climate scenarios and periods. Subplots display PCA results for (**top-left**) the Baseline (1989–2018), (**top-middle**) SSP2-4.5 near-term (2030–2059), (**top-right**) SSP2-4.5 long-term (2070–2099), (**bottom-left**) SSP5-8.5 near-term (2030–2059), and (**bottom-right**) SSP5-8.5 long-term (2070–2099) scenarios. Points are colored by climate zone (red: Zone I, blue: Zone II, green: Zone III) and sized by nitrate leaching (kg·ha^−1^). The variance explained by each principal component (PC1 and PC2) is labeled on the corresponding axes. Red arrows represent the loading vectors of mean annual precipitation (mean_prec) and mean daily temperature (mean_temp), with their loading coefficients annotated alongside.

The principal component analysis (PCA) results are shown in Figure 2. The spatial variation in nitrate leaching is primarily regulated by two independent climate dimensions—temperature and precipitation—and the regulatory effects of climate factors show significant differences across scenarios.

As shown in the figure, the first two principal components (PC1 and PC2) together explain 100% of the total variance across all scenarios, with PC1 as the core explanatory dimension and PC2 as the secondary regulatory dimension. Under the Baseline scenario for the 1989–2018 period, PC1 explained 52.11% of the total variance, and PC2 explained 47.89%. Under the SSP5-8.5 scenario for the 2070–2099 period, the explanatory power of PC1 increased to 60.21%, while PC2 decreased to 39.79%, indicating that the dominant role of temperature was further enhanced. In terms of factor loadings, PC1 was positively correlated with mean annual temperature (λ = 0.707) across all scenarios, and PC2 was positively correlated with annual cumulative precipitation (λ = 0.707), indicating that the regulatory pathways of temperature and precipitation on nitrate leaching remained stable.

The distribution of stations exhibited clear regional heterogeneity. In the Baseline period, stations in Zone I were mainly distributed along the PC1 axis (temperature axis), while stations in Zone III tended to be distributed along the PC2 axis (precipitation axis). This distribution pattern persisted under future scenarios. Furthermore, Pearson correlation analysis showed that nitrate leaching in Zone I had the strongest correlation with mean annual temperature, while nitrate leaching in Zone III had the strongest correlation with precipitation indicators.

The trends in nitrate leaching varied significantly across scenarios. Under the SSP2-4.5 scenario, the mean nitrate leaching was slightly lower than that in the baseline period, while under the SSP5-8.5 scenario, it showed a significant increasing trend, increasing by 12.1% during 2070–2099 compared with the Baseline period, and by 21.8% compared with the SSP2-4.5 scenario for the same period. At the regional scale, the increase in nitrate leaching during 2070–2099 under the SSP5-8.5 scenario was most pronounced in Zone III, reaching the highest among all regions. Atmospheric CO_2_ concentration was also significantly positively correlated with nitrate leaching.

### 2.2. Spatiotemporal Response of Soil Nitrogen Dynamics

#### 2.2.1. Sensitivity of Net N Mineralization to Climate Warming

**Figure 3:**
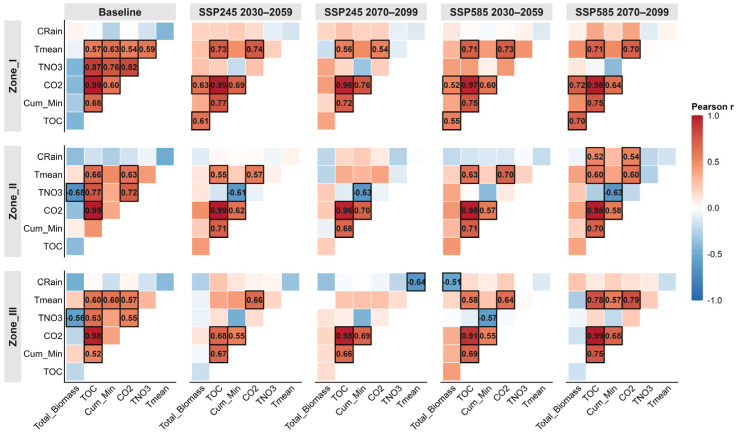
Pearson correlation heatmaps of total biomass vs. environmental/soil variables across three zones (I–III) under five scenarios (Baseline, SSP245/SSP585 2030–2059/2070–2099). Variables: TOC (Total Organic Carbon), Cum_Min (Cumulative N mineralization), CO_2_ (CO_2_ concentration), TNO_3_ (Total nitrate N), Tmean (mean temperature), CRain (cumulative rainfall). Color scale: Pearson *r* (−1 to 1; red = positive, blue = negative), with significant values labeled.

The correlations among climate-soil-crop system indicators are shown in Figure 3. These correlations exhibit high sensitivity to future scenarios, with rising temperature being a key driver accelerating soil organic nitrogen mineralization.

Core indicators within the system showed clear synergistic relationships. Total organic carbon (TOC) exhibited the strongest positive correlation with cumulative nitrogen mineralization (Cum_Min) (r = 0.85, *p* < 0.01), followed by total biomass with TOC (r = 0.78, *p* < 0.01).

The increase in cumulative nitrogen mineralization under the SSP5-8.5 long-term scenario was significantly higher than under the SSP2-4.5 scenario. Under the SSP5-8.5 scenario, the correlation coefficient between mean annual temperature (Tmean) and total nitrate nitrogen (TNO_3_) reached 0.68 (*p* < 0.01), representing a 30.8% increase by comparison with the baseline period. Under the SSP5-8.5 scenario, the correlation coefficient between CO_2_ concentration and total biomass reached 0.72 (*p* < 0.01), which was 18.0% higher than that under the SSP2-4.5 scenario.

The correlation among indicators was most stable in Zone II, where the fluctuation range of the correlation coefficient between TOC and Cum_Min was only 0.82–0.87, significantly smaller than that in Zone I (0.75–0.85) and Zone III (0.78–0.86). Meanwhile, the correlation coefficient between mean annual temperature and annual cumulative precipitation in Zone II (r = 0.42) was lower than that in Zone I (r = 0.58) and Zone III (r = 0.63).

#### 2.2.2. Deep-Layer Nitrate Accumulation and Migration

**Figure 4:**
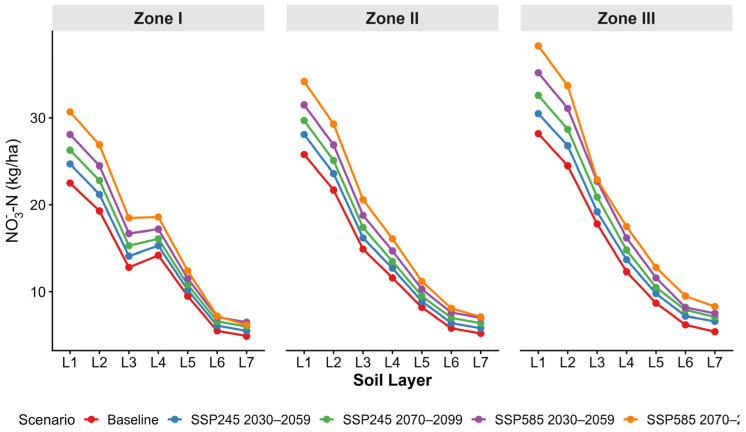
Vertical profiles of soil NO_3_^−^-N (kg/ha) across seven layers (L1–L7) in three zones (I–III) under five scenarios (Baseline, SSP245/SSP585 2030–2059/2070–2099). Lines show NO_3_^−^-N decreasing with soil depth, with maximum contents under SSP585 2070–2099.

The vertical distribution of soil nitrate nitrogen (NO_3_^−^-N) exhibited a distinct “surface enrichment” pattern under all scenarios. However, future climate conditions significantly altered the stability of this pattern, with a substantially increased risk of NO_3_^−^-N accumulation in the 100–150 cm layer, particularly under the SSP5-8.5 scenario (Figure 4).

In the historical baseline period, NO_3_^−^-N storage in the surface 0–20 cm layer accounted for approximately 60% of the total storage in the 0–100 cm profile, and the highest surface accumulation was observed in Zone III. Concentrations decreased stepwise with depth, with sub-100 cm storage representing only a small fraction of the surface values. This general pattern persisted under future scenarios, but the degree of surface enrichment weakened. Under SSP5-8.5 for 2070–2099, the proportion of NO_3_^−^-N in the 0–20 cm layer decreased by 3.5–9.2% relative to the baseline, while storage in the 100–150 cm layer increased markedly.

The 100–150 cm soil layer was used as the primary indicator of deep-layer nitrogen migration because it represents the first depth interval below the main rooting zone where accumulating nitrate signals downward transport. In the baseline period, NO_3_^−^-N storage in this layer was 5.2 kg ha^−1^ in Zone I, 5.5 kg ha^−1^ in Zone II, and 5.8 kg ha^−1^ in Zone III. Under SSP5-8.5 long-term (2070–2099), storage increased to 6.7 kg ha^−1^ (+28.8%) in Zone I, 7.6 kg ha^−1^ (+38.2%) in Zone II, and 8.9 kg ha^−1^ (+53.4%) in Zone III. The largest absolute and relative increases occurred in Zone III, consistent with its higher precipitation and greater precipitation variability under this high-emission scenario. The vertical gradient between surface and deep layers also intensified: the difference in NO_3_^−^-N storage between 0 and 20 cm and 100–150 cm increased by >40% compared with the historical baseline under SSP5-8.5-2070s, reflecting the continued downward displacement of nitrate.

### 2.3. System Productivity and Environmental Impacts Under Adaptive Management

#### 2.3.1. Interspecific N Facilitation and Biological N Fixation (BNF)

The proportion of soybean nitrogen derived from biological nitrogen fixation (BNF) declined consistently with increasing nitrogen application rate across all climate scenarios. Under the Baseline, the BNF proportion ranged from 20.1 to 29.1% under low nitrogen to 11.0–18.6% under high nitrogen, with the highest fixation proportions observed at the earliest sowing date (S1, 19 May). Compared with the Baseline, BNF proportions were 1.2–4.8% lower under SSP2-4.5 and 2.3–7.1% lower under SSP5-8.5, with the sowing date effect remaining consistent across scenarios.

The combined contribution of BNF and soil nitrogen mineralization to total system nitrogen uptake moved in the opposite direction, increasing with nitrogen application rate. Across all scenarios, this combined contribution accounted for 91.0–95.6% of total system nitrogen uptake, with absolute values rising from approximately 383–433 kg ha^−1^ under low nitrogen to 421–477 kg ha^−1^ under high nitrogen in the Baseline, and reaching up to 491–1012 kg ha^−1^ under future scenarios.

**Figure 5:**
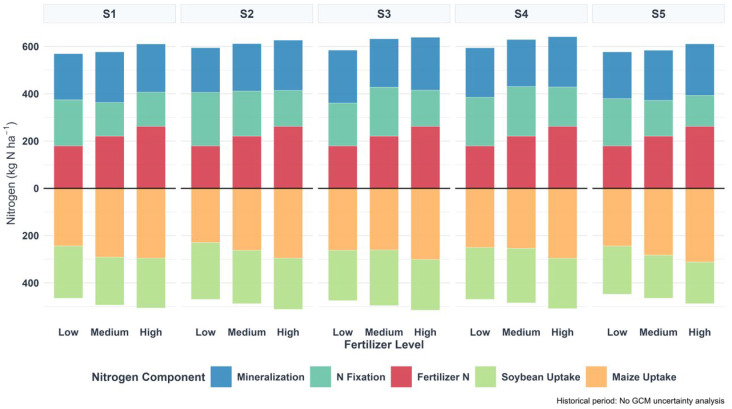
Nitrogen (N) budget components across 5 planting dates (S1–S5) and 3 fertilizer levels (Low, Medium, High) during the historical baseline period (no GCM uncertainty analysis, unit: kg N ha^−1^).

**Figure 6:**
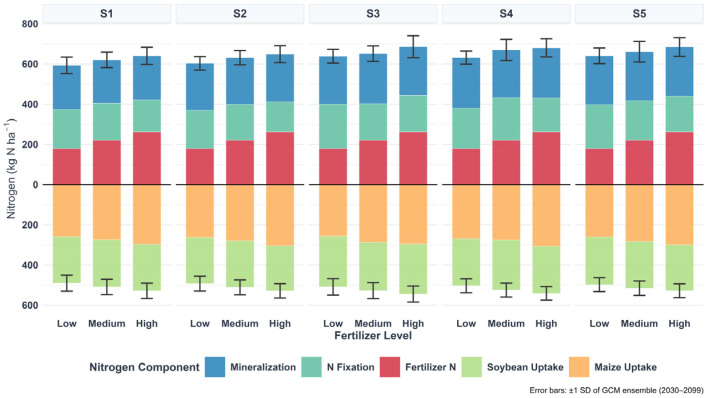
Nitrogen (N) budget components across 5 planting dates (S1–S5) and 3 fertilizer levels (Low, Medium, High) under the SSP2-4.5 scenario during 2030–2099. Error bars represent ±1 SD of the GCM ensemble (unit: kg N ha^−1^).

Total system nitrogen uptake increased steadily with nitrogen application rate, from 771 to 823 kg ha^−1^ under low nitrogen to 949–995 kg ha^−1^ under high nitrogen in the Baseline, with further increases under both future scenarios. The 19 May sowing date consistently showed the highest total nitrogen uptake, whereas the 7 June sowing date showed greater nitrogen use efficiency when considered together with yield outcomes. Detailed nitrogen budget components are presented in Figure 5 and Figure 6 and Supplementary Figure S1.

#### 2.3.2. System Nitrogen Use Efficiency (NUE) and Yield Stability

Partial factor productivity of nitrogen (PFPN) decreased with increasing nitrogen application rate across all scenarios. Under high nitrogen, PFPN averaged 22.1% lower than under medium nitrogen, while nitrate leaching increased by more than 35%, revealing a sharp efficiency–environment trade-off.

Under SSP2-4.5, the combination of the S3 sowing date (7 June) and medium nitrogen performed best, with PFPN reaching 28.9–30.8 kg kg^−1^ across zones, nitrate leaching controlled at 38.5–45.2 kg ha^−1^, and total system yield stable at 16,280–16,600 kg ha^−1^. Under SSP5-8.5, the optimum shifted to S2 (28 May) with medium nitrogen, where PFPN was 25.8–27.2 kg kg^−1^ and total yield was maintained at 16,180–16,430 kg ha^−1^, while the increase in nitrate leaching relative to the baseline was limited to 22.6%. The full PFPN dataset is shown in Supplementary Figure S2.

#### 2.3.3. Optimizing Yield-Environment Trade-Offs

**Figure 7:**
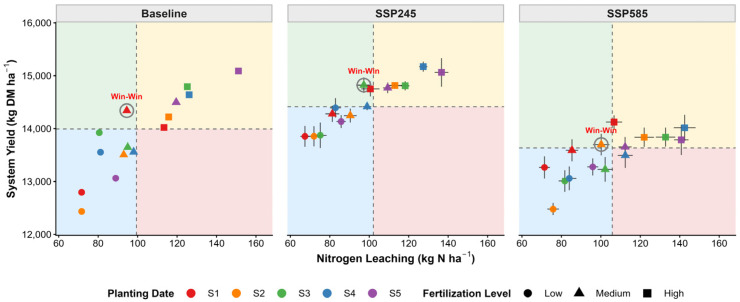
Yield-nitrate leaching trade-off diagram. Scatter plots illustrating the trade-off between system dry matter (DM) yield (kg DM ha^−1^) and nitrate leaching (kg N ha^−1^) across 5 planting dates (S1–S5) and 3 fertilization levels (low, medium, high) under the baseline period, SSP2-4.5, and SSP5-8.5 climate scenarios. The win-win zone (high yield, low nitrate leaching) is highlighted, with error bars representing ±1 standard deviation (SD) of the GCM ensemble.

When all treatment combinations were evaluated on the yield–nitrate leaching plane, they distributed along a clear negative spectrum from high yield–high nitrate leaching to low yield–low nitrate leaching (Figure 7). Using the win–win threshold defined in Section 4.4.3, the combination of S3 sowing (7 June) and medium nitrogen under SSP2-4.5 fell within the target zone. Compared with the conventional farmer treatment (S3 + high nitrogen), this strategy reduced nitrate leaching by approximately 30% while limiting yield loss to less than 2%. Under SSP5-8.5, the S2 sowing (28 May) with medium nitrogen exhibited similar performance, with yield reduction below 3% and nitrate leaching reduced by 25.4%.

Regionally, these strategies maintained stable performance across all three climatic zones, with yield fluctuations below 5% and nitrate leaching controlled substantially below levels observed under high-nitrogen treatments. Based on this dual-objective evaluation, the optimal strategies identified were “S3 + medium nitrogen” under SSP2-4.5 and “S2 + medium nitrogen” under SSP5-8.5.

## 3. Discussion

### 3.1. Mechanistic Linkage Between Precipitation and Deep N Migration

The marked increase in nitrate nitrogen (NO_3_^−^-N) accumulation in the 100–150 cm soil layer under SSP5-8.5 reflects a synergistic “supply–transport” mechanism that is intensified in intercropping systems. On the supply side, warming accelerates soil organic nitrogen mineralization, a relationship consistently demonstrated by Li et al. [26], who linked elevated temperatures to increased urease and protease activities, and by Yang et al. [27], whose DNDC simulations confirmed that warming significantly enhances soil nitrogen mineralization rates. This pattern is consistent with the interpretation that warming-enhanced microbial activity may accelerate mineralization. On the transport side, the substantial increase in precipitation variability under SSP5-8.5 creates conditions favoring episodic deep percolation. This is consistent with the finding of Ke et al. [28] that nitrate leaching increases markedly when daily precipitation exceeds 30 mm, and with field observations by Ning et al. [29] in North China Plain maize systems identifying frequent heavy precipitation events as the primary driver of deep nitrate migration.

What distinguishes this study from previous work on monoculture systems [30,31,32,33] is the coupling of these two drivers within a single growing season. Warming accelerates nitrogen mineralization early in the season, creating a pool of surplus nitrate before peak crop uptake; subsequent high-intensity precipitation events may then mobilize this pre-accumulated nitrate downward. The spatial complementarity of maize and soybean root systems can enhance nitrogen uptake in surface layers, but this interception capacity likely diminishes below 100 cm, leaving deep nitrate vulnerable to further nitrate leaching [34,35]. Previous studies attributing nitrate leaching mainly to excessive nitrogen application or short-term extreme precipitation [30,31,32,33] have not fully captured this coupled supply–transport dynamic.

Regionally, this mechanism produced a distinct spatial gradient: Zone I showed the strongest temperature–leaching coupling, reflecting its larger warming amplitude; Zone III showed the strongest precipitation–leaching coupling, consistent with its greater increase in precipitation variability. This spatial differentiation aligns with the finding of Duan et al. [35] that interspecific root interactions in intercropping systems alter water and nitrogen resource utilization patterns across environments. These results suggest that nitrate leaching control in intercropping systems cannot rely on uniform management prescriptions but must account for the dominant climate driver in each zone.

### 3.2. Synergistic Effects of Sowing Date and N Management on Climate Resilience

The synergistic regulation of sowing date and nitrogen fertilizer can balance yield and nitrate leaching risk in intercropping systems, and different climate scenarios require different optimal combinations. In terms of the fundamental principles of nitrogen regulation, under baseline conditions, the proportion of soybean BNF under low and medium nitrogen treatments was significantly higher than under high nitrogen treatment. This result is consistent with studies by Kou et al. [36] and Villwock et al. [37], showing that medium nitrogen application alleviates the inhibition of soybean nodule nitrogen fixation by high nitrogen, avoiding the “nitrogen inhibition” effect. Meanwhile, the effect of sowing date adjustment on the proportion of BNF in this study aligns with findings by Chen et al. [38], indicating that optimizing sowing date allows crops to avoid high-temperature stress during critical growth stages and better match light, temperature, and water resources.

Unlike existing studies, the core innovative conclusion of this study lies in revealing the significant differences in optimal management combinations under different climate scenarios. Previous studies have mostly focused on optimizing under a single climate scenario [39] or a single management measure [40,41], without addressing the yield-environment dual-objective trade-off of “sowing date + nitrogen fertilizer” under CMIP6 dual scenarios. Through quantitative analysis, this study clearly shows that under the SSP2-4.5 scenario, the combination of the S3 sowing date (7 June) and medium nitrogen treatment achieved the highest partial factor productivity of nitrogen and the lowest nitrate leaching. Under the SSP5-8.5 scenario, however, the optimal combination shifted to the S2 sowing date (28 May) with medium nitrogen treatment. The reason for this difference is that under the SSP5-8.5 scenario, the warming amplitude was significantly higher than that under the SSP2-4.5 scenario, resulting in greater high-temperature stress intensity, necessitating an earlier sowing date to avoid the inhibition of soybean BNF activity by high temperatures during flowering. Zhang et al. [42] found in cotton studies that late sowing combined with nitrogen reduction could improve nitrogen use efficiency without sacrificing yield. This study further extends this principle to intercropping systems and clarifies the specific sowing date thresholds under SSP2-4.5 and SSP5-8.5 scenarios.

Furthermore, the synergistic regulation of sowing date and nitrogen fertilizer in this study enabled the system to maintain stable yield under reduced nitrogen conditions. Under baseline conditions, the combined contribution of BNF and mineralization under medium nitrogen treatment accounted for the majority of total nitrogen uptake, achieving an interspecific division of labor where “maize absorbs soil nitrogen and soybean fixes atmospheric nitrogen.” This finding echoes the conclusion of Kou et al. [43], who observed that optimized row configuration in drip-irrigated intercropping systems could enhance nitrogen transport efficiency. However, the innovation of this study lies in synergistically optimizing sowing date phenological adaptation with nitrogen fertilizer physiological complementarity rather than regulating a single factor.

### 3.3. Practical Implications for Sustainable Agriculture in the NCP

The deep-layer soil nitrogen migration patterns revealed in this study provide key technical support for groundwater protection under the “water and nitrogen limitation” policy in the North China Plain (NCP). In terms of nitrate leaching control effectiveness, the optimal strategies proposed in this study can control nitrate leaching substantially below traditional high-nitrogen treatments. This reduction effect is consistent with existing studies: Ning et al. [29] confirmed that water and fertilizer optimization can reduce nitrate leaching in maize fields in the NCP; Wang et al. [44] found through HYDRUS model simulations that synergistic water and nitrogen regulation can reduce deep-layer nitrate leaching; Shen et al. [45] also pointed out that deep-layer soil nitrogen accumulation is a core risk source for groundwater pollution.

Compared with existing studies, the practical innovation of this study lies in refining the general recommendation of “nitrogen reduction and optimal sowing” [46] into a precise regulation scheme tailored to different climate scenarios and zones. Previous studies have mostly been short-term, single-point field experiments [47,48] that did not account for the spatial heterogeneity of future climate evolution. In contrast, this study, based on long-term simulations across 42 meteorological stations and three climate zones, proposes actionable strategies for differentiated zoning and scenario-specific customization: In Zone I, where nitrate leaching has the strongest correlation with temperature, the strategy focuses on alleviating nitrogen mineralization imbalance caused by warming; in Zone III, where nitrate leaching has the strongest correlation with precipitation, the strategy emphasizes controlling deep-layer nitrate leaching caused by extreme precipitation. Regional adaptability analysis showed that the win-win strategies maintained yield stability and controlled nitrate leaching across all three zones. This zone-specific adaptability provides an operational technical paradigm for agricultural non-point source pollution control in the NCP, aligning closely with the policy orientation of “zoning-based management” in agriculture [49].

Additionally, this study confirms that medium nitrogen application can achieve nitrogen reduction without compromising yield, consistent with the policy direction of reducing fertilizer use while increasing efficiency in the region. Zhang et al. [42] similarly found in cotton studies that nitrogen reduction could improve nitrogen use efficiency while maintaining yield; Hu et al. [50] also demonstrated in rice systems that nitrogen reduction combined with biofertilizer could maintain stable yield and reduce nitrogen losses. This study further extends this principle to the maize-soybean intercropping system and clarifies the sowing date adaptation schemes under different climate scenarios.

### 3.4. Uncertainties and Future Research Directions

While the APSIM model demonstrated satisfactory performance in capturing long-term water and nitrogen cycling trends, several sources of uncertainty warrant consideration. First, the model does not currently incorporate algorithms that explicitly represent the physiological impacts of transient extreme events. For instance, heatwaves can reduce pollen viability and alter nitrogen allocation patterns in ways not fully captured by current crop models [51], and flooding-induced root hypoxia can temporarily suppress nitrogen uptake [27]. In this study, the model was driven by daily climate data and did not explicitly resolve sub-daily extreme precipitation events or short-duration heatwaves. Consequently, the predicted outcomes primarily reflect the systemic effects of long-term baseline climate shifts and may underestimate the instantaneous risks posed by individual extreme events. This limitation is consistent with the uncertainty analysis of Moitzi et al. [52] in wheat systems.

The importance of improving the representation of extreme events in climate impact assessments is increasingly recognized. Zhang et al. [53] emphasized that major drought events in monsoon transition zones are governed by complex interactions among atmospheric circulation patterns, land–atmosphere coupling, and multi-factor synergies operating across spatial and temporal scales, underscoring the need for process-based representation of extreme events in impact models. Similarly, recent catchment-scale research has demonstrated that extreme hydrological events can abruptly alter soil nutrient stoichiometric balances, with cascading effects on nutrient losses to water bodies [54]. Incorporating such event-scale dynamics into next-generation agroecosystem models would improve the reliability of nitrate leaching projections under the most extreme future scenarios.

Second, this study fixed the intercropping row configuration at 2:4 and did not investigate the effects of alternative spatial arrangements. Studies have shown that row ratios significantly alter interspecific competitive relationships and resource allocation patterns [55,56]. Third, some soil nitrogen transformation parameters were retained at default values without region-specific calibration, which may introduce additional uncertainty in the absolute magnitude of nitrate leaching estimates [57], similar to the parameterization challenges identified by Zong et al. [57] in crop rotation systems.

Future research should proceed in three interconnected directions. First, coupling extreme weather impact modules into crop models is essential, with particular attention to heatwave effects on reproductive processes [58] and heavy rainfall effects on preferential flow and deep percolation. Second, the optimization framework should be expanded to include additional management levers such as row configuration [55,56], irrigation regime [59], and soil amendments including biochar [60], which have shown promise in reducing nitrogen losses. Third, multi-model ensemble simulations [61] combined with microbial mechanism studies [62] would provide a more comprehensive understanding of nitrogen cycling under future climates from multiple perspectives. The long-term effects of elevated CO_2_ on crop residue decomposition and soil nitrogen mineralization, as noted by Mattoo et al. [63], also merit investigation through long-term field experiments integrated with modeling. Ultimately, validating the adaptive strategies proposed in this study across different soil types, climate zones, and crop variety combinations through coordinated field experiments will be critical for their practical deployment in the North China Plain and similar semi-humid intensive agricultural regions worldwide.

## 4. Materials and Methods

### 4.1. Study Site and Soil Characterization

The North China Plain (NCP) is the second largest plain in China. The region has a continental monsoon climate, characterized by warm and humid conditions from April to September, with most annual precipitation concentrated between June and August. The soils in the study area are predominantly fluvo-aquic soils developed through river alluviation, with a loam texture. In the typical cultivated soil layer, the field capacity ranges from 24% to 30%, bulk density from 1.30 to 1.45 g·cm^−3^, and organic carbon content from 6.0 to 12.0 g·kg^−1^. This soil type is the main cultivated soil for the maize-soybean intercropping system [64].

**Figure 8:**
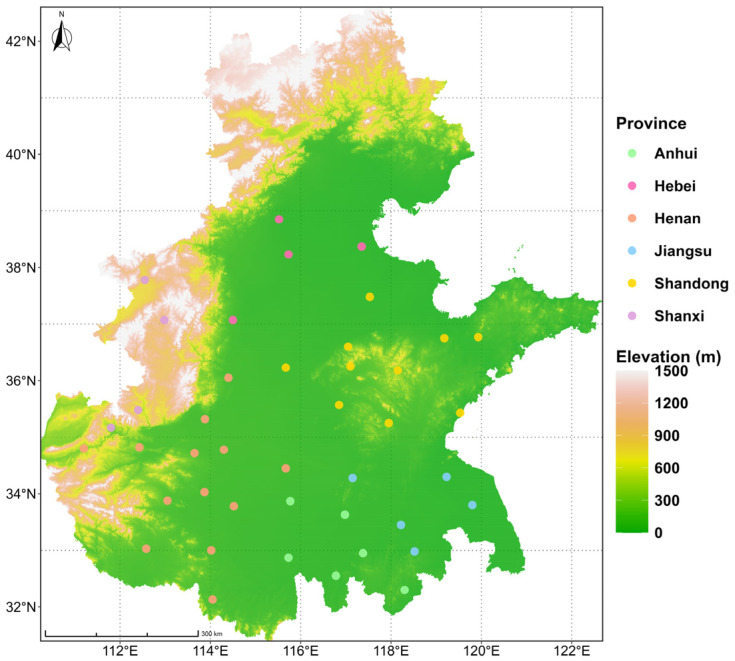
Spatial distribution of 42 meteorological stations in the North China Plain (NCP). Different colors indicate corresponding provinces, and background color gradient reflects elevation spatial variation of the study area.

In this study, 42 evenly distributed meteorological stations were selected from the areas suitable for maize-soybean intercropping cultivation in the North China Plain, including central and southern Hebei, Henan, central and western Shandong, and northern Jiangsu and Anhui (Figure 8). Based on climatic differences, the study area was divided into three regions: I. Northern Warm Temperate Zone (36° N–39° N), II. Central Warm Temperate Zone (34° N–36° N), and III. Southern Warm Temperate Zone–Subtropical Transition Zone (32° N–34° N). These divisions characterize the heterogeneity of climate, crop varieties, and biophysical soil conditions across regions. Considering the climatic differentiation characteristics, three representative meteorological stations were selected for APSIM calibration and validation: Raoyang (38.23° N, 115.73° E) in Zone I, Xinxiang (35.32°N, 113.88°E) in Zone II, and Nanyang (33.03° N, 112.58° E) in Zone III.

In this study, the soil profile data corresponding to the selected stations were all derived from the Harmonized World Soil Database (HWSD) version 2.0. The soil hydraulic parameters and initial nitrogen pool data were directly extracted from the profile data of the corresponding regions in this database without additional localization adjustments to ensure the reliability of the basic data for nitrogen cycle simulation. The soil hydraulic parameters include bulk density (BD), saturated water content (SAT), field capacity (DUL), and wilting point (LL); the initial soil nitrogen pool includes nitrate nitrogen, ammonium nitrogen, and organic nitrogen contents. In combination with the physical and chemical characteristics of the typical cultivated soil layer in the region, the soil organic carbon and background nitrogen values in the study area were also determined. The soil physical parameters of different soil layers at the three representative stations are presented in Supplementary Table S1.

### 4.2. Climate Data and Scenario Construction

#### 4.2.1. Historical Observations

The baseline historical daily scale climate data employed in this study cover the period 1961–2018 and include four core variables: daily solar radiation, maximum temperature, minimum temperature, and precipitation. All data were obtained from the China Meteorological Administration (CMA, https://data.cma.cn), with complete and continuous time series that meet the requirements of the APSIM model for long-term daily-scale climate inputs. For the daily solar radiation (Rad) required by the APSIM model, the Angström–Prescott formula was employed to estimate it based on sunshine duration, as follows:

(1) $$Rad=\left(a+b\frac{n}{N}\right)R_{a}$$

where *Rad* represents daily solar radiation (*Rad*, MJ m^−2^d^−1^). The variables *n* and *N* denote the actual and theoretical daily sunshine durations, respectively. The coefficients *a* and *b* were calibrated against solar radiation observations at the selected stations.

#### 4.2.2. CMIP6 Future Projections

In this study, future climate simulation data from 20 CMIP6-based Global Climate Models (GCMs) were retrieved from https://esgf-node.llnl.gov/search/cmip6/ (accessed on 1 February 2026). A statistical downscaling model was employed to downscale the monthly gridded climate data of the 20 GCMs to a daily scale for the 42 selected stations. Statistical downscaling models are now widely used in assessing the impacts of future climate change. For this assessment, two typical Shared Socioeconomic Pathway (SSP) scenarios were selected: SSP2-4.5 and SSP5-8.5. SSP2-4.5 represents a moderate CO_2_ emission scenario, under which global CO_2_ concentrations are projected to peak around 2040, gradually decline in the late 21st century, and stabilize by the end of the century, consistent with global annual CO_2_ emission trends. SSP5-8.5 represents a high CO_2_ emission scenario, designed to reflect a pathway with no strict emission reduction but strong adaptation to climate change. Under this scenario, global annual greenhouse gas emissions continue to increase throughout the 21st century; both scenarios will experience rising temperatures, with the maximum temperature reached by the end of the century. In the APSIM model, CO_2_ concentration affects crop growth and development by regulating the transpiration factor (TF), radiation use efficiency (RUE), and leaf nitrogen concentration, thus serving as an important parameter in the model. The formulas for calculating annual CO_2_ concentrations under the SSP2-4.5 and SSP5-8.5 scenarios are as follows:

(2) $$CO_{2,\mathrm{SSP}245}=62.044+\frac{34.002-3.8702Y}{0.24423-1.1542Y^{2.4901}}+0.028057{\left(Y-1900\right)}^{2}\;+0.00026827{\left(Y-1960\right)}^{3}-9.2751\times 10^{-7}{\left(Y-1910\right)}^{4}-2.2448$$

(3) $$CO_{2,\mathrm{SSP}585}=757.44+\frac{84.938-1.537Y}{2.2011-3.8289Y^{-0.45242}}+2.4712\times 10^{-4}{\left(Y+15\right)}^{2}\;+1.9299\times 10^{-5}{\left(Y-1937\right)}^{3}+5.1137\times 10^{-7}{\left(Y-1910\right)}^{4}$$

where, *Y* denotes the year from 2030 to 2100 (*Y* = 2030, 2031, 2032, …, 2100). This study simulates one historical period (1989–2018) and two future periods (2030–2059 and 2070–2099). A constant CO_2_ concentration of 350 ppm was used for the simulations during the 1989–2018 period.

#### 4.2.3. GCM Selection and Bias Correction

Due to differences in initial and boundary conditions, spatial resolution, and other factors among GCMs, uncertainties exist in future climate change predictions, and simulation accuracy may vary by region [65]. Evaluating GCM simulation accuracy based on historical meteorological data helps identify GCMs with higher simulation accuracy [66]. To balance simulation efficiency and representativeness of results while adapting to the biophysical differences across the three climatic zones of the North China Plain, a three-stage quantitative screening was conducted for the initially obtained 20 CMIP6 GCMs. The historical observational data used for screening included daily maximum temperature, daily minimum temperature, precipitation, and solar radiation from 42 meteorological stations in the North China Plain from 1961 to 2018, which were downscaled to daily scale data.

In the first stage, the S-score was used to quantify the simulation accuracy of each GCM for four core climate factors (maximum temperature, minimum temperature, precipitation, and radiation). S-values closer to 1 indicate higher simulation accuracy, calculated using the following formula:

(4) $$S=\frac{4\times {\left(1+R\right)}^{2}}{{\left(\frac{\sigma_{m}}{\sigma_{0}}+\frac{\sigma_{0}}{\sigma_{m}}\right)}^{2}\times {\left(1+R_{0}\right)}^{2}}$$

where *σ*_m_ and *σ*_0_ are the standard deviations of simulated and observed values, respectively, *R* is the spatial correlation coefficient between them, and *R*_0_ is the maximum *R* value among all GCMs.

Based on this, a weighted composite score was constructed (maximum temperature S-score × 0.3, minimum temperature S-score × 0.3, precipitation S-score × 0.25, solar radiation S-score × 0.15) to integrate the synergistic effects of multiple climate factors on simulation results. Based on the station-level composite scores across the entire region, the top 9 high-performance GCMs were preliminarily selected from the 20 GCMs.

In the second stage, a nitrogen dynamics-specific score was incorporated, including three meteorological indicators closely related to crop nitrogen utilization: heavy precipitation frequency matching (frequency of daily precipitation ≥ 25 mm events), proportion of suitable temperature days (proportion of days with daily maximum temperature within the 15–30 °C suitable range), and drought stress degree (maximum consecutive days without effective precipitation). The final composite score was constructed (baseline score accounting for 85%, nitrogen dynamics score accounting for 15%), with weights assigned to the crop growing season from May to October and enhanced weights for simulation accuracy during the critical growth period from July to August. Thus, comprehensive quantification of model performance was achieved by calculating the final composite scores for stations across the entire region.

**Figure 9:**
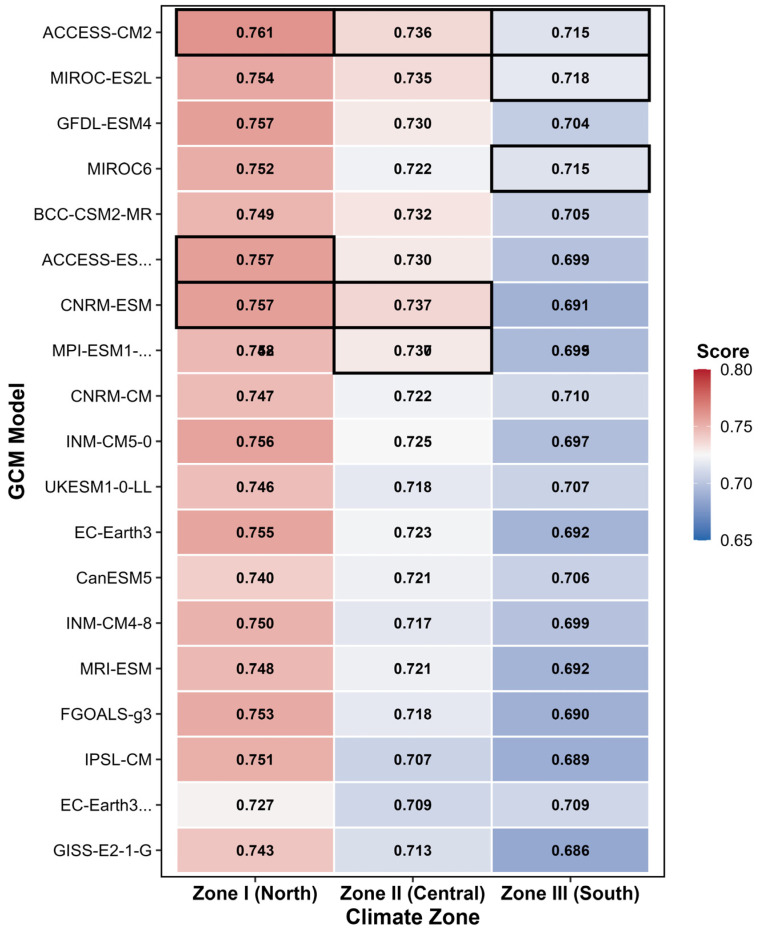
Comprehensive scoring results of final GCM screening. The optimal GCMs for each climatic zone are as follows: Zone I: ACCESS-CM2, ACCESS-ESM1-5, CNRM-ESM; Zone II: ACCESS-CM2, MPI-ESM1-2-LR, CNRM-ESM; Zone III: ACCESS-CM2, MIROC-ES2L, MIROC6.

**Figure 10:**
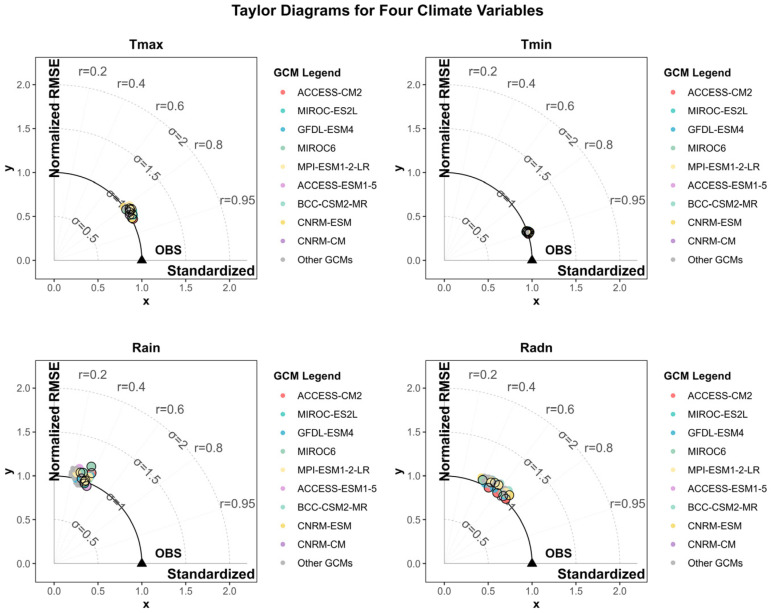
Taylor diagrams of core meteorological elements. The figure presents Taylor diagrams for daily maximum temperature (Tmax), daily minimum temperature (Tmin), precipitation (Rain), and solar radiation (Radn), respectively. By integrating statistical indicators such as correlation coefficient (R), standardized standard deviation (σ), and root mean square error (RMSD), it intuitively compares the agreement between simulations from different CMIP6-GCMs and station observational data. The closer the simulation point is to the observation point (OBS) and the higher the match of standard deviation, the better the model simulation performance.

In the third stage, the composite scores were ranked by climate zone (Figure 9). The Taylor diagram (Figure 10) was then used to visualize the evaluation results, integrating the correlation coefficient, root mean square error, and standard deviation. The top 3 representative GCMs were ultimately selected for each zone. The Taylor diagram results showed that the selected models exhibited high spatial correlation with observational data, good standard deviation matching, and significantly better simulation performance than the other models.

The final selected GCMs had composite scores ranging from 0.715 to 0.761 and offered the following core advantages: (1) high simulation accuracy for key climate variables such as temperature and precipitation in the study area, with composite scores in the high range; (2) coverage of diverse model structures and physical parameterization schemes, effectively reducing the uncertainty associated with single-model simulations; (3) adaptation to the meteorological characteristics of different climate zones, enabling accurate characterization of regional climate heterogeneity; and (4) simultaneous consideration of climate simulation during the crop growing season and characterization of meteorological factors relevant to nitrogen dynamics, fully meeting the driving requirements of the APSIM model.

To further eliminate systematic biases between GCM-simulated data and historical observational data, the Multivariate Bias Correction using N-dimensional probability density function transform (MBCn) method was applied to correct the selected GCM data. This method simultaneously corrects the marginal distributions of individual climate variables and the correlations between variables, making the simulated results more consistent with the actual meteorological variation characteristics of the North China Plain (Supplementary Figures S3–S5) and ensuring that the corrected climate data can directly drive the APSIM model for crop growth simulations.

### 4.3. APSIM Model Configuration and Evaluation

#### 4.3.1. Model Description and Intercropping Module

APSIM (Agricultural Production Systems Simulator) is a process-based agroecosystem simulation model that accurately characterizes crop growth and development, soil material transport, and interspecific resource competition under monoculture and intercropping systems [67]. This study used APSIM version 7.10 to construct a maize-soybean intercropping simulation system. Through a modular coupling mechanism, the model dynamically quantifies interspecific competition and complementarity for aboveground light, belowground water, and nitrogen in intercropping systems.

The APSIM MicroClimate module incorporates the core computational methods for canopy light interception and interspecific light competition, which serve as the key module for simulating aboveground light resource competition in intercropping systems. In this study, the intercropping system was established and operated using the built-in Canopy interface and the Intercrop Management script in the Manager folder. The characteristic parameters for light competition between the two crop canopies were uniformly defined and configured through external cultivar parameter files.

The model simulates belowground interspecific competition for water and nitrogen resources through the coupling of the SoilWat (soil water module), SoilN (soil nitrogen module), and SoilArbitrator (soil arbitration module). The SoilWat module simulates soil water infiltration, evaporation, percolation, and root water uptake based on the 10-layer soil profile configuration, crop root distribution, and root water uptake coefficient (KL), quantifying the spatial competition between maize and soybean roots for available soil water. The SoilN module couples nitrogen transformation processes such as soil organic nitrogen mineralization, nitrification, and denitrification, simulating the transport and uptake of soil nitrate nitrogen (NO_3_^−^) and ammonium nitrogen (NH_4_^+^), and quantifying the competitive uptake of inorganic nitrogen by the two crops. The SoilArbitrator module adopts RootBiomassWeighted as the resource allocation rule for water and nitrogen, dynamically allocating available soil water and nitrogen resources based on the real-time root biomass ratio of maize and soybean, realistically reflecting the dynamic nature of belowground resource competition in intercropping systems.

The model’s built-in Soybean crop module includes a biological nitrogen fixation (BNF) function, which simulates the fixation of atmospheric nitrogen by soybean rhizobia, quantifies the nitrogen resources supplemented by soybeans through BNF, and characterizes the nitrogen complementarity effect in maize-soybean intercropping systems, effectively reducing interspecific nitrogen competition intensity and aligning with the nitrogen cycling characteristics of intercropping systems in actual production.

#### 4.3.2. Crop Cultivar Calibration and Validation

This study employed APSIM version 7.10 to simulate the maize–soybean intercropping system, with model calibration following the sequential priority of phenology, biomass, and yield. Three representative stations and three sets of commonly used cultivars in the North China Plain (NCP) were selected. Based on published field experimental observation data for maize and soybean cultivars, localized calibration of cultivar parameters was conducted using a trial-and-error method. The calibrated cultivar parameters for maize and soybean are listed in Supplementary Tables S2 and S3. The detailed literature data are provided in Supplementary Table S4. Additionally, independent validation of the model was further performed using measured yield data from maize-soybean intercropping plots at the Xinxiang Experimental Station of the Chinese Academy of Agricultural Sciences in 2025 (representative station). Three indicators—coefficient of determination (R^2^), root mean square error (RMSE), and mean absolute error (MAE)—were selected to quantitatively evaluate the simulation accuracy of maize and soybean models during calibration and validation stages.

The results showed that during the model calibration phase, simulated yields of maize and soybean agreed well with observed values across all calibration and validation datasets. Key performance metrics are summarized in Table 1, and detailed scatter plots with density distributions are provided in Supplementary Figures S6–S8.

#### 4.3.3. Nitrogen Cycle Verification

The calculation formulas and rate parameters for the core processes of soil nitrogen cycling (mineralization, nitrification, denitrification, ammonia volatilization, and nitrate leaching) followed the model’s default values. This parameter system has been previously widely validated and applied in nitrogen cycle simulations of maize-soybean intercropping systems in the North China Plain (NCP) [68]. This study did not adjust the core parameters of soil nitrogen cycling but only conducted localized calibration for parameters related to crop nitrogen uptake and allocation. Measured nitrate nitrogen data from the 0–20 cm soil layer in 2025 at the Xinxiang Experimental Station of the Chinese Academy of Agricultural Sciences, combined with soil inorganic nitrogen observation data from representative stations such as Raoyang and Nanyang, were used to independently validate the model’s nitrogen dynamics simulation performance.

The model satisfactorily captured soil NO_3_^−^-N dynamics across all three sites, irrigation treatments, and crop growth stages. Model performance metrics (coefficient of determination, R^2^); root mean square error, RMSE; mean absolute error, MAE) are as follows: at Nanyang, R^2^ = 0.75, RMSE = 3.22 kg/ha, and MAE = 2.56 kg/ha; at Raoyang, R^2^ = 0.70, RMSE = 3.40 kg/ha, and MAE = 2.93 kg/ha; and at Xinxiang, R^2^ = 0.73, RMSE = 3.32 kg/ha, and MAE = 2.86 kg/ha. These results confirm the model’s ability to reliably simulate soil nitrate-nitrogen dynamics under the study conditions. All detailed scatter plots comparing simulated and observed values are provided in Supplementary Figure S9.

### 4.4. Simulation Experiment Design

#### 4.4.1. Intercropping Configuration

**Figure 11:**
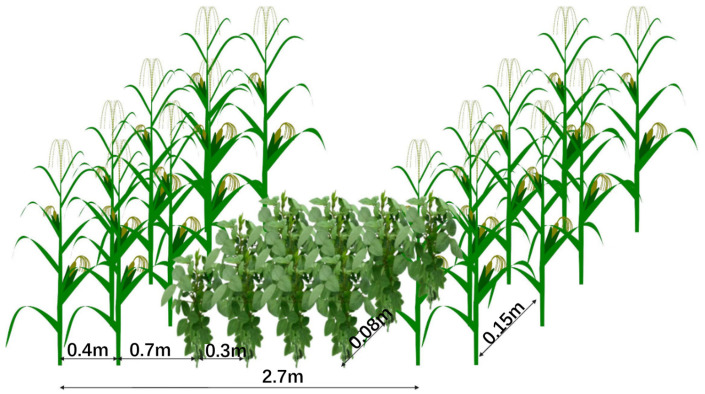
Schematic diagram of maize-soybean strip intercropping with row and plant spacing parameters (unit: m).

This study adopted the maize-soybean 2:4 strip intercropping system, which is widely promoted in the North China Plain (NCP). The maize row spacing was set at 40 cm, the soybean row spacing at 30 cm, and the spacing between the maize strip and the soybean strip was set at 70 cm. The maize plant spacing was set at 15 cm, and the soybean plant spacing at 8 cm (Figure 11). Since APSIM automatically allocates space based on the input parameters, indirectly reflecting the 2:4 row ratio, the intercropping pattern was achieved in the APSIM intercropping module by combining planting density and row spacing. The final input planting density for maize was 4.94 plants·m^−2^, and for soybean it was 18.52 plants·m^−2^.

#### 4.4.2. Management Variables

To develop agronomic management strategies adapted to future climate change, this study set two core adaptive variables for the maize-soybean intercropping system: sowing date and nitrogen management.

For sowing date regulation, based on the traditional summer maize planting rhythm in the North China Plain (NCP) and the characteristics of future climate variability, five gradient sowing dates were established (S1, 19 May; S2, 28 May; S3, 7 June; S4, 16 June; S5, 22 June). The sowing window covered late May to late June. This gradient sowing date design effectively broadens the crop growth adaptation range, fully covers the changes in the growth window caused by temperature and precipitation fluctuations under future climate scenarios, and avoids the adverse effects of extreme climate stresses such as high temperature during flowering and periodic droughts on crop growth.

For nitrogen management, based on the nitrogen demand patterns of crops under future climate conditions and the goal of controlling soil nitrate leaching, this study established three nitrogen application gradients (low, medium, and high) for maize. The medium N rate for maize (480 kg urea ha^−1^, equivalent to 220.8 kg pure N ha^−1^ based on 46% N content in urea) was selected to fall within the locally recommended N application range of 180–240 kg N ha^−1^ for summer maize in the North China Plain [69,70]. This rate was split into basal, stem elongation, and flowering applications as detailed in Table 2 to match maize N uptake dynamics. The low N rate (390 kg urea ha^−1^, 179.4 kg N ha^−1^) and high N rate (570 kg urea ha^−1^, 262.2 kg N ha^−1^) were designed to bracket this recommended range. Soybeans received a uniform basal application of 60 kg urea ha^−1^ (equivalent to 27.6 kg N ha^−1^) as a starter fertilizer to support early vegetative growth before the onset of active biological nitrogen fixation. This practice is consistent with the typical recommendation of 30–75 kg urea ha^−1^ for soybean in the region. This approach matches the nitrogen uptake requirements of maize and soybean at different growth stages and, by appropriately regulating the total nitrogen application rate and distribution ratio, thus reduces the risk of nitrate leaching caused by excessive nitrogen application, balancing yield improvement and farmland nitrogen environmental safety.

For comparative analysis, the conventional farmer management was defined as sowing on 7 June (S3) with the high N rate (570 kg urea ha^−1^, equivalent to 262.2 kg N ha^−1^), which reflects the typical practice of applying 250–270 kg N ha^−1^ to summer maize in the North China Plain.

#### 4.4.3. Scenario-Specific Optimization

This study established three consecutive simulation periods: the historical baseline (1989–2018), near-term future (2030–2059), and long-term future (2070–2099), to compare the intercropping system’s response under different climate backgrounds. The sowing trigger condition was set as cumulative rainfall of no less than 20 mm between days 140 and 170 of each year, with a uniform sowing depth of 40 mm, consistent with traditional field sowing practices in the North China Plain (NCP). The model was driven by daily-scale meteorological data, including solar radiation, daily maximum temperature, daily minimum temperature, and precipitation. All simulations were conducted under rainfed conditions [71].

The simulation process was divided into two stages. In the first stage, based on historically observed meteorological data, the system performance of different intercropping cultivar combinations in the three climatic zones during the baseline period (1989–2018) was simulated. In the second stage, the model was driven by the three optimal GCMs selected from the 20 CMIP6 GCM ensemble under two Shared Socioeconomic Pathway (SSP) scenarios (SSP2-4.5 and SSP5-8.5) to simulate crop yield and phenological changes under different combinations of sowing dates and nitrogen application treatments during the near-term (2030–2059) and long-term (2070–2099) future periods. A total of 8190 independent simulations were conducted using a high-performance computing platform, establishing a simulation database including multiple scenarios, management measures, and regions.

A “win-win” management strategy was defined as one that reduces yield by less than 5% relative to the baseline scenario while achieving a reduction in nitrate leaching greater than 20%. The 5% yield threshold corresponds to the typical range of statistically non-significant yield variation reported in field experiments in the North China Plain, while the 20% nitrate leaching reduction target aligns with nitrogen management benchmarks for groundwater quality protection in intensive agricultural regions [29,72].

## 5. Conclusions

1. With future climate warming and changes in precipitation spatiotemporal distribution (particularly under the SSP5-8.5 high-emission scenario), the “water-nitrogen supply-demand mismatch” in the North China Plain’s maize-soybean intercropping system will continue to intensify. This will lead to a substantial increase in the risk of deep-layer (100–150 cm) nitrate accumulation, with increases of 28.8% (Zone I), 38.2% (Zone II) and 53.4% (Zone III) relative to the baseline period, and a 12.1% increase in total nitrate leaching, posing a long-term and serious threat to regional groundwater safety. The formation of this risk pattern is closely related to the advance of nitrogen mineralization induced by climate warming, the shortening of the crop nitrogen uptake window, and the deep percolation triggered by extreme precipitation events.
2. The strong compensatory capacity of soybean biological nitrogen fixation (BNF) is a key ecological mechanism buffering intercropping systems against the impacts of climate fluctuations. Under low and medium nitrogen conditions, the synergistic contribution of BNF and mineralization accounted for 49.4–58.3% of total nitrogen input, effectively compensating for spatiotemporal fluctuations in exogenous nitrogen supply. However, excessive nitrogen application significantly inhibits this mechanism, reducing the proportion of soybean BNF by 27.6–33.9%, leading to a decline in system nitrogen utilization resilience and a sharp increase in environmental emission risks.
3. Implementing adaptive management centered on “phenology-nutrient” dual regulation is key to achieving a “yield-environment” win-win outcome. Pareto optimal analysis integrating yield stability and low nitrate leaching objectives recommends the following: under the SSP2-4.5 moderate warming scenario, adopt the S3 sowing date (7 June) with medium nitrogen application (total pure nitrogen application rate of 220.8 kg·ha^−1^); under the SSP5-8.5 high warming scenario, adopt the S2 sowing date (28 May) with medium nitrogen application (total pure nitrogen application rate of 220.8 kg·ha^−1^). This synergistic strategy achieves both risk mitigation and efficiency enhancement by optimizing the spatiotemporal matching of crop growth periods with climate resources and leveraging physiological complementarity in interspecific nitrogen utilization.
4. This synergistic optimization strategy not only offsets 30–35% of negative climate impacts, limiting system yield losses to within 3%, but also reduces nitrate leaching risk by 25–30%. Moreover, it provides a quantitative decision-making reference and technical paradigm for similar semi-humid intensive agricultural regions worldwide to leverage traditional intercropping wisdom in addressing long-term climate evolution and achieving sustainable agricultural development. Its core logic aligns with the agricultural intensification theory of “precise matching of resource supply and demand,” offering empirical support for constructing climate-smart agricultural management systems.

## Figures and Tables

**Table 1:** Calibration and validation results of maize and soybean models at three stations.

Crop	Station	Stage	*n*	*R* ^2^	RMSE	MAE
Maize	Raoyang	Calibration	21	0.840	811.8	677.9
Maize	Xinxiang	Calibration	15	0.924	633.4	553.0
Maize	Nanyang	Calibration	15	0.938	907.2	803.6
Soybean	Raoyang	Calibration	11	0.787	138.4	112.2
Soybean	Xinxiang	Calibration	10	0.959	113.8	88.1
Soybean	Nanyang	Calibration	11	0.793	219.2	207.4
Maize	Raoyang	Validation	9	0.804	493.3	390.8
Maize	Xinxiang	Validation	9	0.779	669.2	507.3
Maize	Nanyang	Validation	9	0.746	292.5	231.4
Soybean	Raoyang	Validation	9	0.766	85.8	68.6
Soybean	Xinxiang	Validation	9	0.553	85.6	69.4
Soybean	Nanyang	Validation	9	0.787	49.3	37.2

**Table 2:** Nitrogen fertilization regimes for maize.

Fertilization Gradient	Total Urea Application Rate (kg·ha^−1^)	Sowing Stage (kg·ha^−1^)	Stem Elongation Stage (kg·ha^−1^)	Anthesis Stage (kg·ha^−1^)
Low N	390	120	150	120
Medium N	480	150	180	150
High N	570	180	210	180

## Data Availability

The original contributions presented in the study are included in the article; further inquiries can be directed to the corresponding author.

## Supplementary Materials

The following supporting information can be downloaded at: https://www.mdpi.com/article/10.3390/plants15111753/s1, Figure S1: Nitrogen budget components at five locations under SSP585 across three fertilization levels; error bars = ±1 SD of GCM ensemble (2030–2099). Figure S2: PFPN variations with five sowing dates under baseline, SSP245 and SSP585 for three fertilization rates; error bars = ±1 SD of GCM ensemble. Figure S3: Interannual changes in daily solar radiation (Radn), precipitation (Rain), maximum temperature (Tmax), and minimum temperature (Tmin) under SSP2-4.5 and SSP5-8.5 scenarios during 2030–2059 and 2070–2099 for Zone I. Figure S4: Interannual variations in solar radiation (Radn), precipitation (Rain), maximum temperature (Tmax), and minimum temperature (Tmin) under SSP2-4.5 and SSP5-8.5 scenarios during 2030–2059 and 2070–2099 for Zone II. Figure S5: Interannual variations in solar radiation (Radn), precipitation (Rain), maximum temperature (Tmax), and minimum temperature (Tmin) under SSP2-4.5 and SSP5-8.5 scenarios during 2030–2059 and 2070–2099 for Zone III. Figure S6: Maize yield calibration across three zones with fit indices and observed-simulated yield density distributions. Figure S7: Soybean yield calibration across three zones with validation statistics and yield distribution curves. Figure S8: Model validation of grain yield for six cultivars at three experimental sites; R^2^, NSE, RMSE and MAE annotated for each subplot. Figure S9: Soil nitrate-N validation at Nanyang, Raoyang and Xinxiang; performance metrics (R^2^, RMSE, MAE, nRMSE, ME) inset per site. Table S1: Soil physicochemical properties at three experimental sites across 0–200 cm soil layers. Table S2: Key cultivar parameters of three summer maize varieties for NCP simulation. Table S3: Growth and physiological parameters of three soybean cultivars. Table S4: Detailed literature data for cultivar parameter calibration.

## Abbreviations

The following abbreviations are used in this manuscript:

APSIM	Agricultural Production Systems Simulator
BD	Bulk Density
BNF	Biological Nitrogen Fixation
CMIP6	Coupled Model Intercomparison Project Phase 6
DUL	Field Capacity
GCM	Global Climate Model
LL	Wilting Point
MBCn	Multivariate Bias Correction using N-dimensional probability density function transform
NCP	North China Plain
NH_4_-N	Ammonium Nitrogen
NO_3_-N	Nitrate Nitrogen
PCA	Principal Component Analysis
PFPN	Partial Factor Productivity of Nitrogen
SAT	Saturated Soil Water Content
SOM	Soil Organic Matter
SSP	Shared Socioeconomic Pathway
TN	Total Nitrogen
