# Supplementary material for: Optimizing Sowing Date and Nitrogen Management to Trade Off Yield and Nitrate Leaching in Maize-Soybean Intercropping Under CMIP6 Climate Scenarios in the North China Plain
Source: Plants (Basel). 2026 Jun 4;15(11):1753. doi: 10.3390/plants15111753 (PMC13259372; doi:10.3390/plants15111753)
Supplement: Supplementary file 1 [file plants-15-01753-s001.zip › Table S1-S3.pdf]

Table S1. Soil physical and chemical parameters of representative stations.

Site	depth	BD	SOM	TN	NO_3-N	NH_4-N	LL	DUL	SAT
Raoyang	0–20	1.398	8.27	0.886	24.820	10.637	0.14	0.26	0.422
	20–40	1.418	5.90	0.667	18.688	8.009	0.14	0.26	0.415
	40–60	1.440	4.42	0.469	13.143	5.633	0.14	0.26	0.406
	60–80	1.445	3.49	0.423	11.843	5.076	0.14	0.26	0.405
	80–100	1.476	3.20	0.396	11.076	4.747	0.14	0.26	0.393
	100–150	1.485	2.92	0.369	10.344	4.433	0.14	0.26	0.389
	150–200	1.488	2.41	0.443	12.411	5.319	0.12	0.25	0.388
Xinxiang	0–20	1.400	9.99	1.016	28.453	12.194	0.14	0.27	0.422
	20–40	1.434	6.22	0.708	19.836	8.501	0.15	0.27	0.409
	40–60	1.443	4.50	0.512	14.349	6.150	0.14	0.27	0.405
	60–80	1.456	3.52	0.447	12.516	5.364	0.14	0.26	0.400
	80–100	1.466	3.24	0.416	11.659	4.997	0.14	0.26	0.397
	100–150	1.509	2.93	0.342	9.572	4.102	0.14	0.26	0.381
	150–200	1.525	2.56	0.439	12.294	5.269	0.13	0.26	0.375
Nanyang	0–20	1.373	14.39	1.302	36.467	15.629	0.15	0.29	0.432
	20–40	1.463	7.39	0.785	21.968	9.415	0.17	0.29	0.398
	40–60	1.471	4.99	0.549	15.371	6.587	0.18	0.30	0.395
	60–80	1.492	3.60	0.486	13.612	5.834	0.18	0.30	0.387
	80–100	1.577	3.14	0.466	13.061	5.598	0.18	0.30	0.355
	100–150	1.589	2.92	0.455	12.743	5.461	0.17	0.29	0.350
	150–200	1.652	2.31	0.461	12.915	5.535	0.16	0.28	0.327

Table S2 Summer Maize Variety Parameters for Three Subregions of the North China Plain

Cultivar Parameters	Cultivar		
	JNK728	JD20	ZD958
tt_emergence_to_end_of_juvenile (°C d)	230	220	250
tt_flowering_to_maturity (°C d)	730	750	760
tt_flowering_to_grain_filling (°C d)	100	120	130
photoperiod_slope (°C h^{-1})	14	13	15
grain_filling_rate (mg·$grain^{-1}$·d^{-1})	22	20	25
max_grains_per_ear	680	520	600
plant_height (cm)	230	220	250
Rue(g·MJ^{-1})	1.5	1.6	1.8

Table S3 Soybean Variety Parameters for the Three Subregions of the North China Plain

Cultivar parameters	Cultivar

	HD21	ZH301	ZD1307
tt_emergence_to_juvenile (°C d)	220	220	210
tt_flowering_to_maturity (°C d)	600	550	600
tt_flowering_to_pod_filling (°C d)	300	320	320
photoperiod_slope (°C h^{-1})	18	17	19
grain_filling_rate (mg·$grain^{-1}$·d^{-1})	12	11	13
max_grains_per_plant	60	55	65
plant_height(cm)	100	90	100
n_fixation_rate(g·$plant^{-1}$·d^{-1})	8	7	9
pod_partitioning_fraction	0.75	0.7	0.8
Rue(g·MJ^{-1})	1.7	1.8	1.9
