# Supplementary figures and images for: Optimizing Sowing Date and Nitrogen Management to Trade Off Yield and Nitrate Leaching in Maize-Soybean Intercropping Under CMIP6 Climate Scenarios in the North China Plain
Source: Plants (Basel). 2026 Jun 4;15(11):1753. doi: 10.3390/plants15111753 (PMC13259372; doi:10.3390/plants15111753)

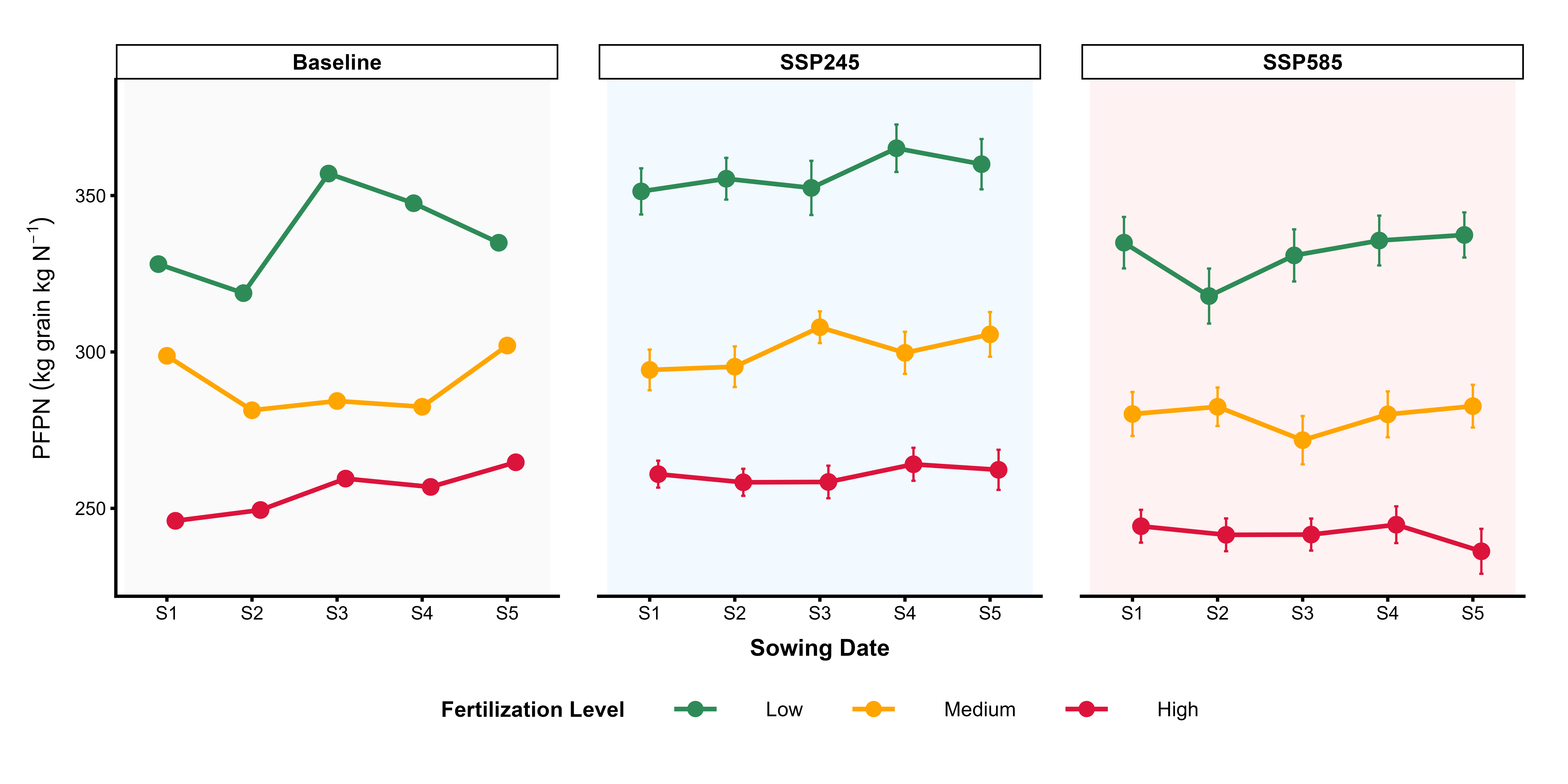

Supplement: Supplementary file 1 [file plants-15-01753-s001.zip › Figure S2.tiff]

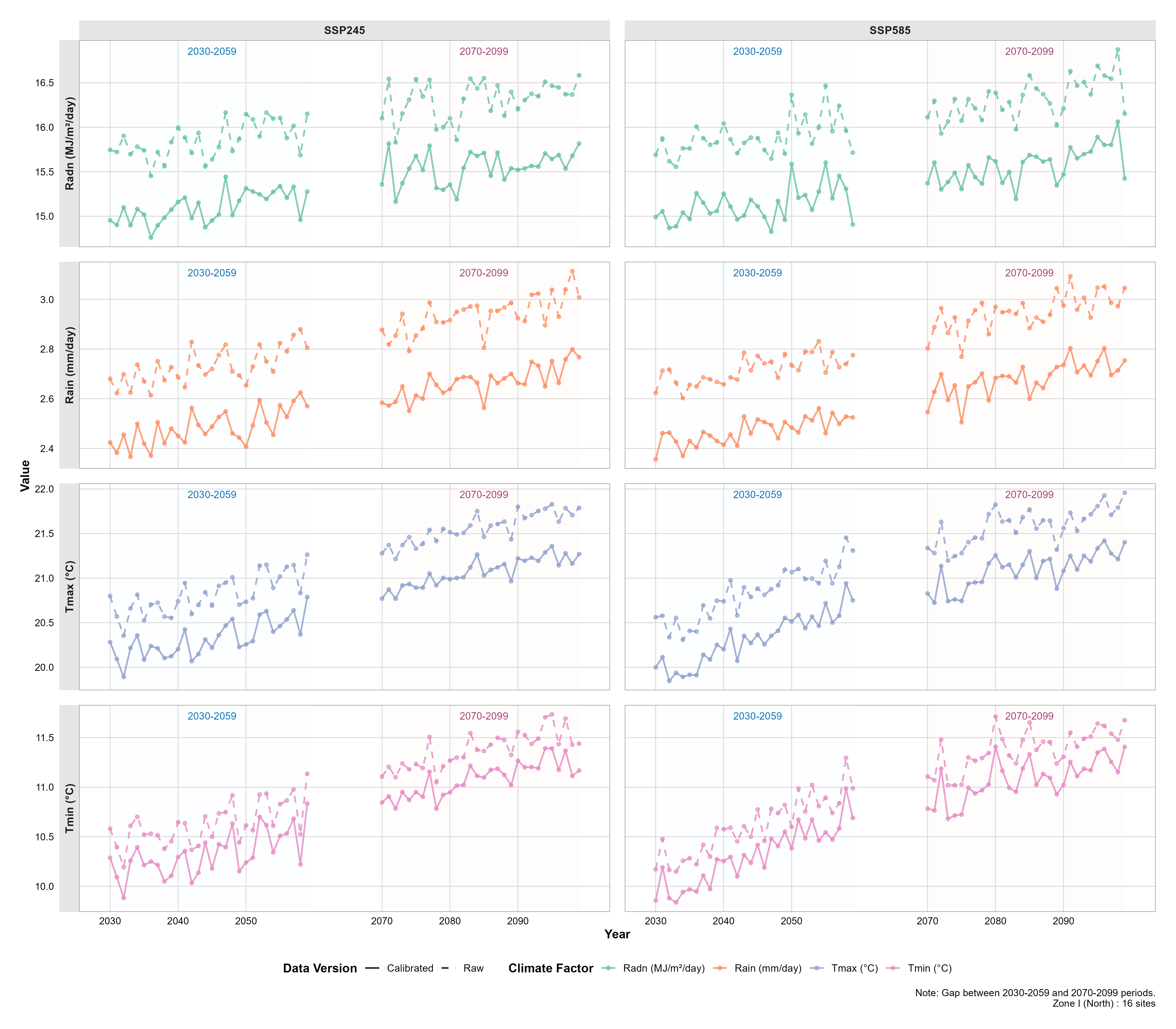

Supplement: Supplementary file 1 [file plants-15-01753-s001.zip › Figure S3.png]

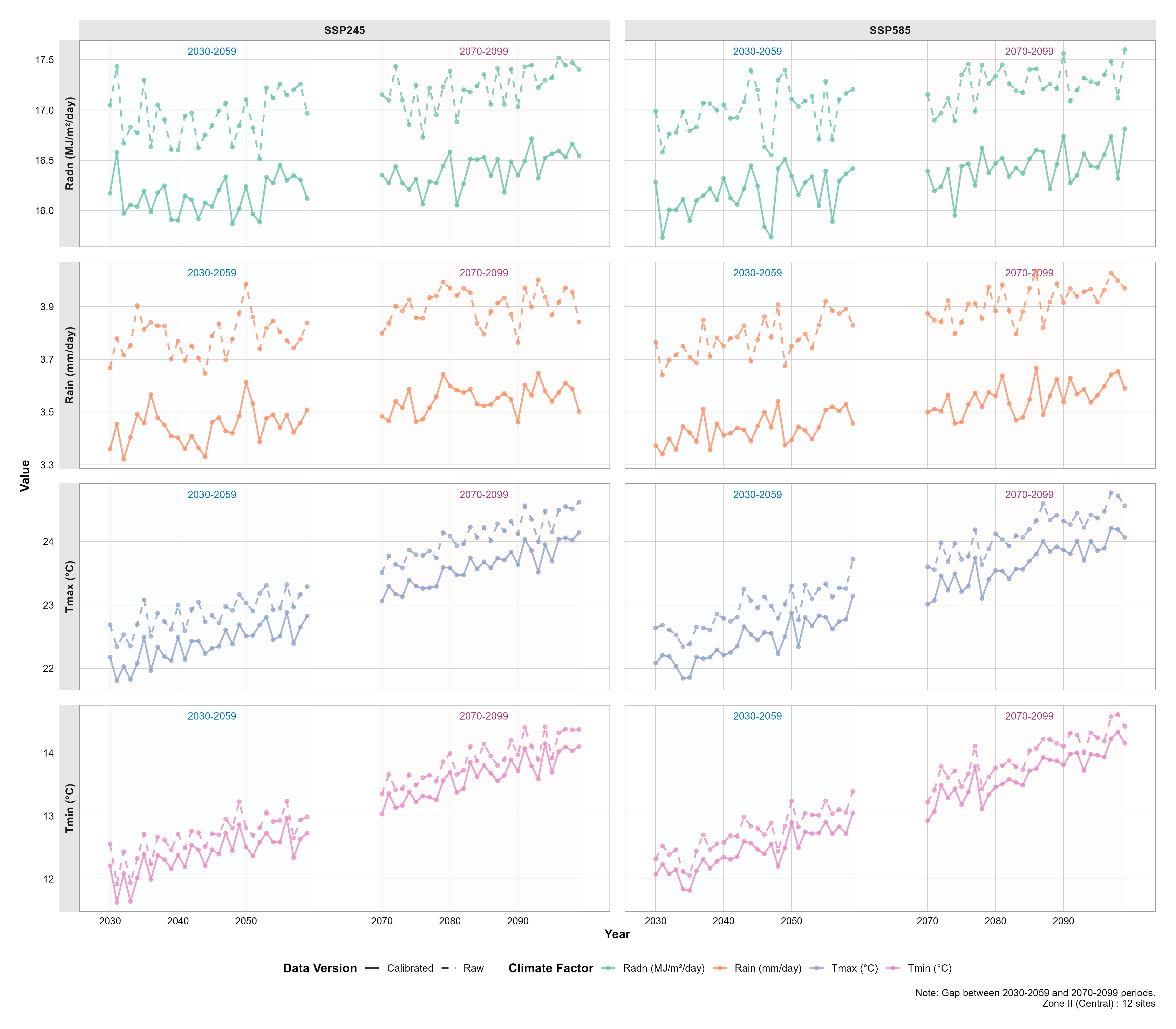

Supplement: Supplementary file 1 [file plants-15-01753-s001.zip › Figure S4.png]

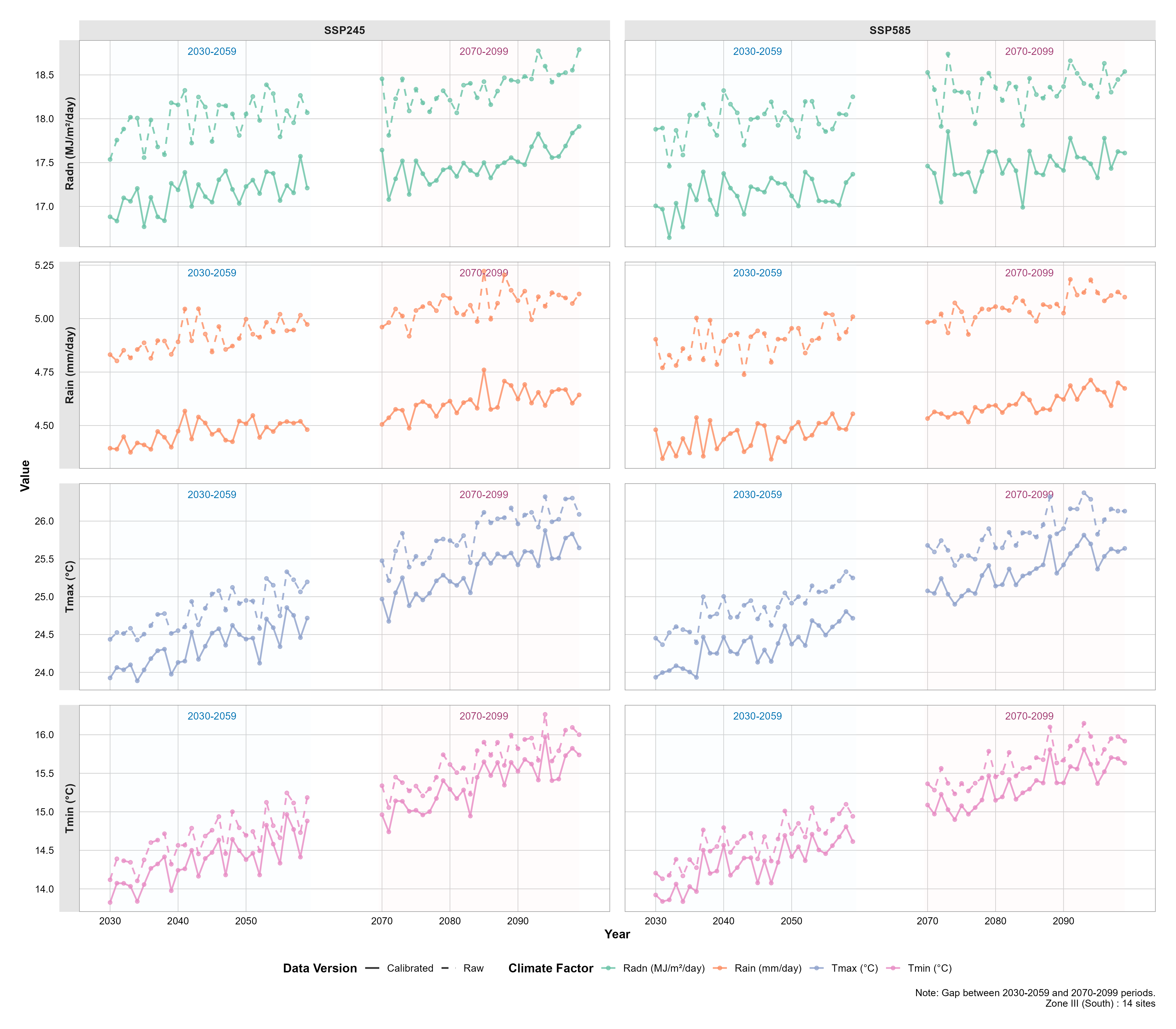

Supplement: Supplementary file 1 [file plants-15-01753-s001.zip › Figure S5.png]

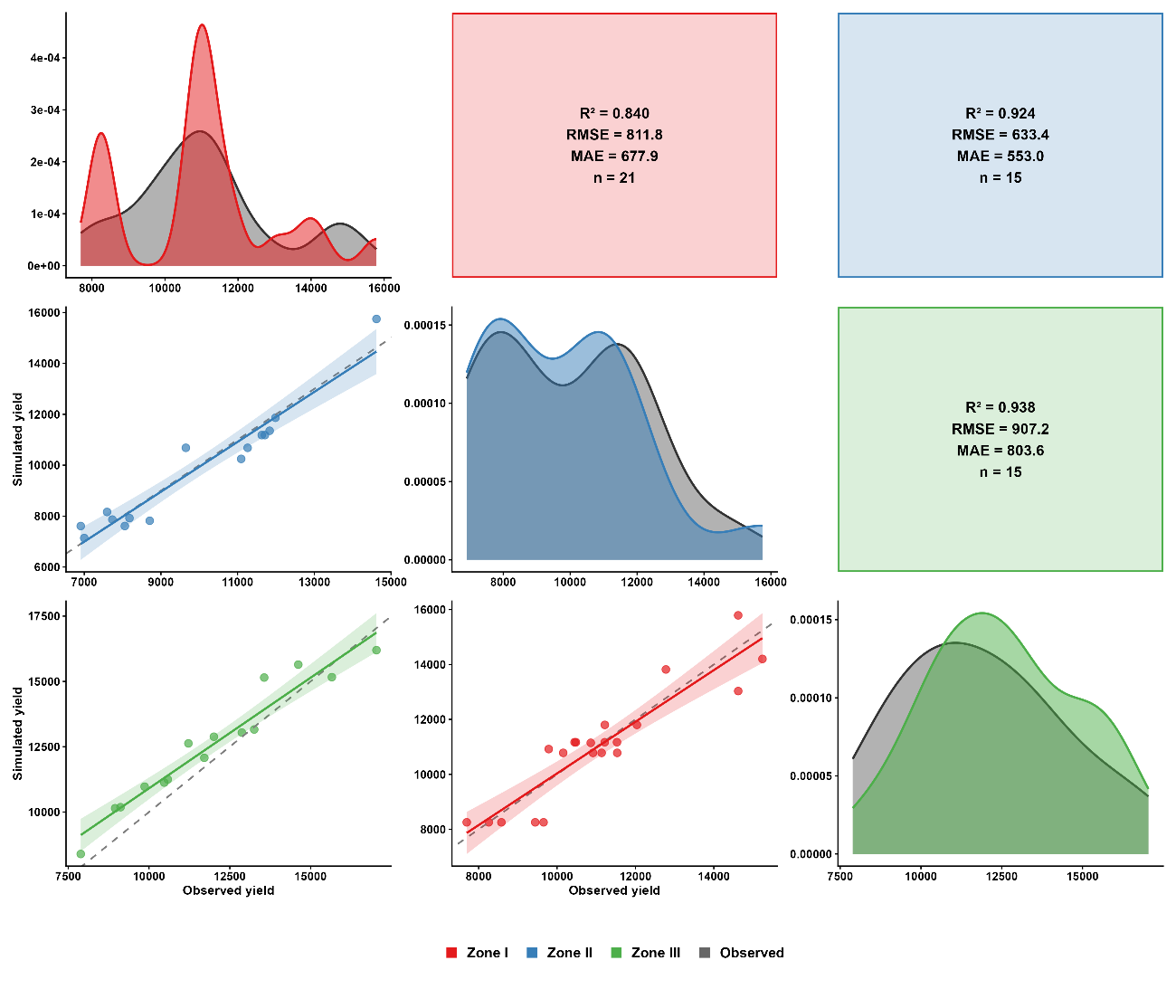

Supplement: Supplementary file 1 [file plants-15-01753-s001.zip › Figure S6.tif]

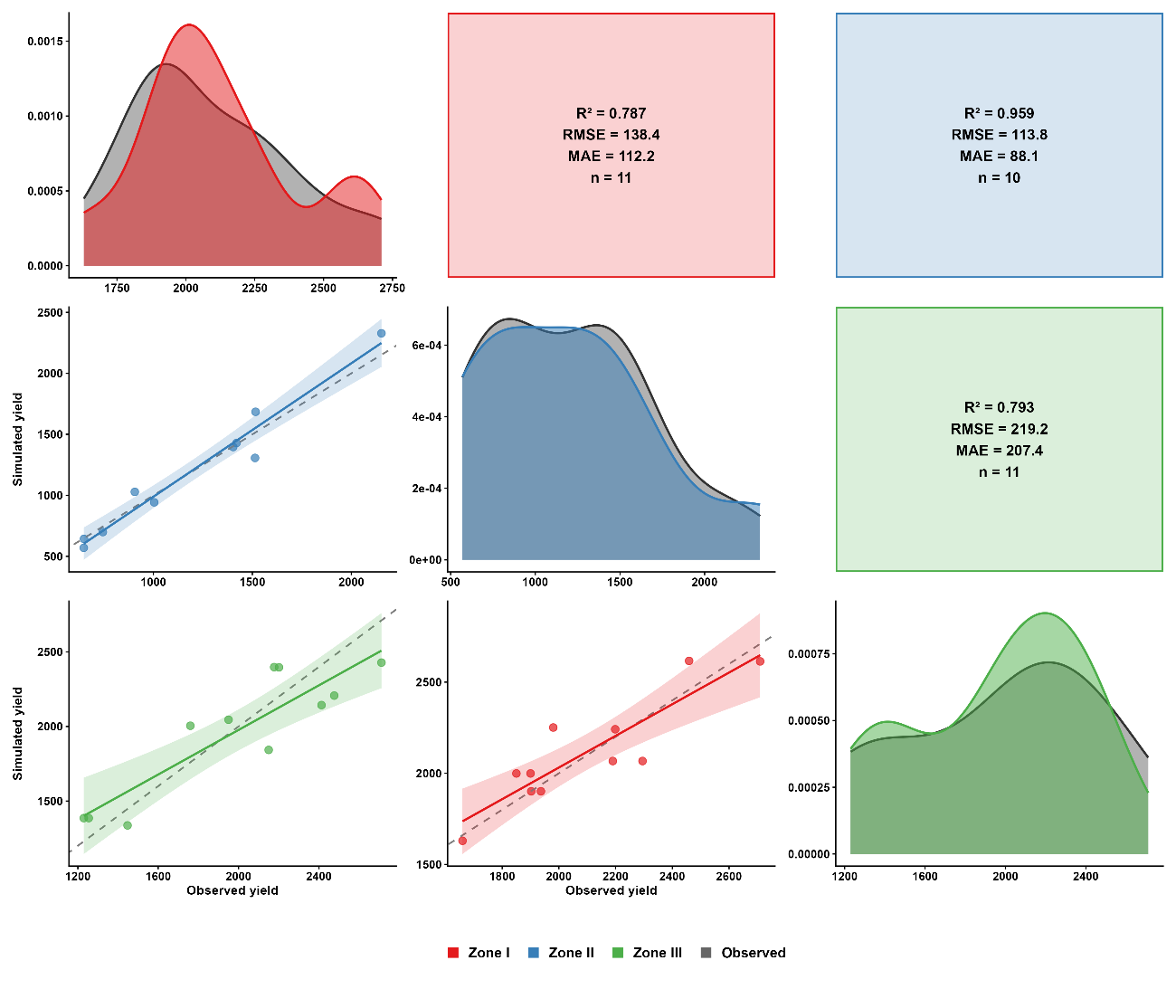

Supplement: Supplementary file 1 [file plants-15-01753-s001.zip › Figure S7.tif]
